# Immunological response of live-captured wild elk (*Cervus canadensis*) to Treponeme-Associated Hoof Disease antigens

**DOI:** 10.3389/fvets.2025.1652577

**Published:** 2026-02-05

**Authors:** Jennifer Wilson-Welder, Kristin Mansfield, Sushan Han, Brock Hoenes, David Alt, Sam Humphrey, Steven C. Olsen

**Affiliations:** 1 Infectious Bacterial Diseases of Livestock Research Unit, National Animal Disease Center, Agricultural Research Service - USDA, Ames, IA, United States; 2 Washington Department of Fish and Wildlife, Spokane Valley, WA, United States; 3 Colorado State University Diagnostic Medicine Center, Fort Collins, CO, United States; 4 Histology and Microscopy Services, National Animal Disease Center, Agricultural Research Service - USDA, Ames, IA, United States

**Keywords:** *Cervus canadensis*, hoof disease, immune response, *Treponema*, wildlife

## Abstract

A severe hoof disease is affecting wild elk (*Cervus canadensis*) in the Pacific Northwest. Causing lameness, tissue ulceration and necrosis, hoof overgrowth, and often loss of the hoof itself, bacteria from the genus *Treponema* are found at the forefront of the lesions. As part of a study evaluating survival and the ability of affected elk to raise young, lymphocytic responses to treponemal antigens were evaluated in live-captured female elk from the endemic area. Serum antibody titers correlated with disease severity and increased antigen-reactive B cells in peripheral blood when compared to healthy or naïve elk. However, we found no evidence that a high antibody titer reduced disease. T lymphocytic responses, CD4+ and CD8+, were mildly proliferative to treponemal antigen, correlating with advanced stages of lesion development. Elk with early-stage lesions also had gamma-delta T cells that proliferated in response to treponeme antigen. Gamma-delta T cells in cattle and sheep have been shown to translocate to the skin preferentially and also have been shown to have a high affinity for spirochete antigens; however, their role in hoof disease of elk or other livestock is not fully understood. In general, initially healthy animals had low lymphocytic responses, indicating that naturally acquired immunity in natural infection is probably rare. Further study is needed to determine the roles of lymphocytes in the protection or perpetuation of these bacteria-driven hoof diseases. In general, immune responses correlated with the severity of disease, with higher responses seen in animals with late-stage disease. No animals were observed with high levels of bacteria responsive immunity in the healthy state, as would be observed if a vaccine or existing immunity was present. Thus, naturally occurring immunity to this disease may be rare. More study is needed to determine the role of immunologically based protection for this and other treponeme-driven hoof diseases.

## Introduction

Free-roaming elk in the US Pacific Northwest are experiencing an increase in severe hoof lesions, with investigations suggesting a bacterial etiology similar to digital dermatitis in livestock. The lesions consist of predominantly neutrophilic infiltrates, with keratinocyte hyperproliferation, necrosis, and loss of skin architecture (1). Bacterial communities are composed of many opportunistic anaerobic species, including *Fusobacterium, Prevotella, Clostridia,* and *Actinobacter*, as well as *Treponema* spp. found at the leading edge of lesions (2). This information has resulted in the condition being termed Treponeme-Associated Hoof Disease (TAHD).

Originally described within elk herds in southwestern Washington State, current reports have suggested endemic disease throughout Washington and Oregon, northern California, western Idaho, and southern British Columbia, Canada (3). TAHD can affect numerous members of a herd and appears to be easily transmitted from animal to animal. Lesions begin as raw, eroded lesions in the interdigital space or along the coronary band (1) (grade 1), progressing to ulcerative necrosis underrunning the hoof horn, causing hoof overgrowth (grade 2), eventually perforating the hoof sole (grade 3), and eventually causing additional keratinocyte proliferation with necrosis, eventually leading to sloughing of the hoof capsule (grade 4). The raw underlying lamina may fill by granulation but more frequently becomes infected, leading to joint sepsis, gangrene, and/or necrosis of the distal joints.

The impact of the high prevalence and rapid spread of TAHD in reducing elk populations was of concern, especially related to the recruitment of young animals needed for herd stability. Thus, a multi-year study was undertaken by the Washington Department of Fish and Wildlife (WDFW) to determine the effect of TAHD on the survival and reproductive capacity of elk within impacted game management units. Additional samples were collected for assays to evaluate the systemic immune response of the affected elk to treponemal antigens. In cattle, there is a locally strong inflammatory response driven by innate immune signaling and keratinocytes, including production of IL1b, upregulation of TLR signaling, and infiltration of pro-inflammatory macrophages (4–6). While a robust antibody response is generated to treponemal antigens in naturally affected cattle (7–14), efforts to elucidate a systemic, adaptive immune, or memory response post-infection have found little evidence (15, 16). Following infection and treatment, a small study showed that dairy cattle had very little CD4+ recall response to treponemal antigens (15). Antibody and cellular responses to other bacteria found in the digital dermatitis lesions do increase with infection; however, since many of these bacteria or closely related species are members of the rumen flora, it is difficult to make clear interpretations (13, 15). Thus, the focus has been on treponemes, which not only dominate the immune response but are at the invasive edge of the lesions. Given the similar underlying bacterial pathogens, with *Treponema* dominant and at the leading edge of the lesions and cellular pathologies (17, 18), TAHD in elk is a natural disease model for other digital dermatitis diseases, such as bovine digital dermatitis and contagious ovine digital dermatitis of small ruminants. The elk hoof disease occurs without management confounding factors like poor hygiene or treatment interventions. The multi-year study offered the opportunity to potentially observe the same set of animals over time and determine if they developed any immune response before disease occurred, if the presence of antibody in the serum was protective, or if there was recovery from disease or survival with hoof disease.

### Study objective

Determine the immunological response in wild elk with and without TAHD lesions in an endemic area, and determine the correlation of immune responses, both cellular and humoral, to the presence or severity of TAHD lesions.

## Materials and methods

### Sample collection

**Figure 1:**
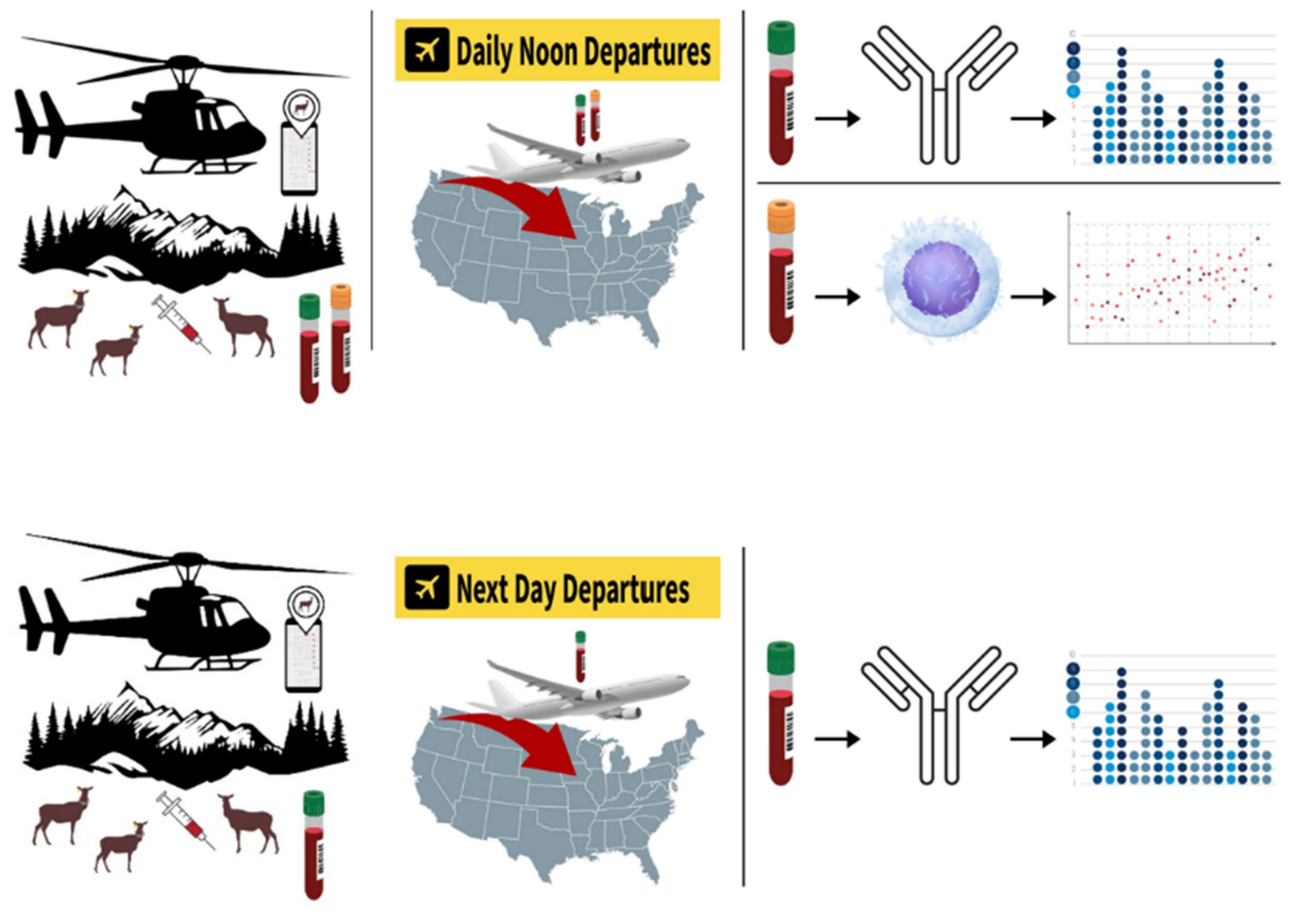
Pictorial diagram of elk capture sampling. After the initial capture, elk were identified to replace deceased TAHD+ or negative, and previously collared elk were relocated by helicopter. If animal was sampled before noon, both serum for antibody ELISA assays and blood for PBMC analysis were collected and sent same day for overnight arrival at lab. If the animal was sampled after noon, then only serum for antibody ELISA was collected and sent with the next day’s samples.

Animals were part of a larger study conducted by the WDFW assessing health factors and survival in female elk in the endemic area. The animal study was approved by the WDFW Health Section. The study was conducted in accordance with the local legislation and institutional requirements. Elk were selected to maintain a study population of approximately 80 radio-collar-fitted female elk of breeding age (>2 years old) with a ratio of 3:1 of TAHD to non-affected animals. Female elk were captured using aerial darting from a helicopter using recommended immobilizing and reversal agents (19). The survey area consisted of 5 game management units in southwest Washington State, which encompass the range of the Greater Mount St. Helen’s elk herd (20). Live captures took place in February 2015, December 2015, December 2016, December 2017, and December 2018. Captured elk were identified using ear-tags and a mortality-sensitive, GPS (Global Positioning System)-equipped radio-collar. Refer to the summary report by B. Hones for further details on the study (20). Feet were scored for the presence of TAHD lesions once captured by experienced personnel according to an established 5-point grading scale (0–4) as previously described (1). During capture, an upper canine tooth was removed to determine age using micro-histological analysis of cementum annuli (Matson’s Laboratory, Milltown, MT) (21). Whole blood for peripheral blood mononuclear cell (PBMC) isolation was collected in heparin tubes, and blood for serum was collected using serum separator vacutainer tubes. Due to shipping logistics, only animals captured before noon, Monday through Thursday, were sampled for immunological studies. Animals sampled after 12:00 or on other days when overnight shipments were not possible were still included in serum analysis and survival data. Tubes were shipped overnight by a commercial carrier to the ARS research laboratory at the National Animal Disease Center (NADC) in Ames, Iowa. Warming packs (Hot Hands™ hand warmers) were included in the shipping boxes to prevent freezing or prolonged chilling during shipment. A schematic is depicted in Figure 1.

Blood samples from naïve or unexposed elk were collected from elk in the Yakima Valley using a similar capture protocol (20). Additional naive blood samples were obtained from elk housed at the NADC, in which no lesions of TAHD have been observed. The total number of animals captured per timepoint, lesion grades, and source for the naïve animals are listed in Table 1.

**Table 1:** Number of female elk captured at each timepoint and TAHD status.

Timepoint	Number of new TAHD+	Number of new healthy (TAHD−)	Number previously collared TAHD+	Number previously collared TAHD−	Number of naïve elk sampled (and source)	Number of samples collected for ELISA	Number of samples collected for PBMCs
Feb 2015	59	18			8, Yakima	74	8
Dec 2015	5	10	20	11	0	46	24
Dec 2016	18	5	15	8	0	42	4
Dec 2017	14	15	6	4	2, NADC	44	24
Dec 2018	20	10	9	7	3, NADC	49	0

### Antigen preparation

*Treponema phagedenis,* strain 4A (22), *Treponema denticola* [(ex Flügge) Chan et al. (ATCC 35405)], *Treponema medium* [Umemoto et al. (ATCC 700293)], and *Treponema pedis* (Evans et al. DSM 18691) were grown in Oral Treponeme Enrichment Broth (Anaerobe Systems, Morgan Hill, CA) to confluence, bacterial cells were harvested and washed by centrifugation, and antigen was prepared from whole-cell sonicates as described previously (16). Briefly, after washing by centrifugation, cells were resuspended in a minimal amount of sterile distilled water. Resuspended cells were frozen at −80 °C overnight and lyophilized. Dried powdered bacterial cells were stored at −20 °C until used to create a 10 mg/mL suspension in sterile phosphate-buffered saline (pH 7.4, PBS). The rehydrated suspension was pulsed for a total of 1 min in 15-s pulsed bursts, resting on ice for 30 s between bursts at 50% power using a probe sonicator. The sonicated solution was placed in a sterile Petri dish and placed in a UV-Stratalinker for 15 min at full power, swirling occasionally to sterilize the whole cell sonicate preparation. Aliquots were frozen at −20 °C until used for antigen stimulation or ELISA. *Fusobacterium necrophorum* subsp. necrophorum (Flugge) Moore and Holdeman (ATCC 25286) and *Porphyromonas levii* (Johnson and Holdeman) Shah et al. (ATCC 29147) were purchased from ATCC and grown on Brucella Agar Plates (Difco) supplemented with 5% defibrinated sheep blood, 1 mL (0.1 g/mL EtOH) Vitamin K1 (w/v) (Sigma), and 1 mL (10 mg/mL) w/v hemoglobin (equine source, Sigma), harvested by gentle scraping, washed 3 times in PBS by centrifugation, and whole cell sonicates prepared as above.

### Enzyme-linked immunosorbent assay (ELISA)

Whole blood collected in serum separator vacutainer tubes was allowed to clot, and serum was separated by centrifugation (Beckman Coulter Avanti J-E with JS-5.3 rotor, 700×*g*, 20 min, 4 °C) and stored at −20 °C. The table of collected samples used in ELISA assays by timepoint and lesion grade is listed in Supplementary Table S1. Bacterial antigens used were an equal mix of *T. phagedenis, T. pedis, T. medium*, and *T. denticola* whole cell sonicates, adjusted to 10 μg protein/mL in PBS, and bound overnight to a 96-well plate (Costar High Binding, Costar) (100 μL/well). Plates were blocked for 4 h at room temperature with 300 μL per well of Pierce Protein-Free Blocking Buffer (ThermoFisher), and then washed once by filling wells with PBS containing 0.05% (v/v) Tween 20 (PBST). Collected serum was serially diluted (1:50 to 1:128,000) and added to plates in duplicate and incubated at 4 °C overnight. Bound antibody was detected by horseradish peroxidase-conjugated donkey anti-goat IgG H&L (KPL, Gaithersburg, MD) (1:10,000 dilution, 100 μL/well), incubated 1 h at 37 °C. The color reaction was developed with SureBlue Reserve TMB Microwell Peroxidase Substrate (KPL-SeraCare), and the reaction was stopped with SureBlue Reserve TMB Stop Solution (KPL-SeraCare). Plates were read at 455 nm. The reported titer is the lowest dilution with optical density equal to or greater than the mean plus two standard deviations of wells containing PBS. The assay was performed by a technician blinded to the elk’s TAHD status. ELISA results were analyzed using GraphPad Prism 9 statistical software, fitting a two-way ANOVA and Dunnett’s multiple comparisons for simple effects within columns to log-transformed (log2) data. Due to the low number of samples for grades 2 and 3, these categories were combined for analysis. Finding no difference within grade across timepoints, an ordinary one-way ANOVA with Tukey’s multiple comparisons was run to determine statistical differences between lesion grades without a variable timepoint.

### PBMC isolation

Whole blood collected in heparinized tubes was diluted 1:2 with sterile PBS, and PBMCs were isolated by density gradient centrifugation (Histopaque, Sigma, density 1087). Isolated PBMCs were labeled with 10 nM Cell Trace Violet (Invitrogen) for proliferation assays before being cultured in the presence of 25 μg/mL bacterial whole cell sonicates, 10 μg/mL pokeweed mitogen (positive stimulation control), or medium alone (background or no stimulation) at 2.5 × 10^6^ cells/mL in a round-bottom 96-well plate at 37 °C in triplicate, 5% CO_2_ for 5 days. Cell culture media was RPMI 1640 supplemented with 10% fetal calf serum, 10 mM L-glutamine, Anti-Anti (Gibco), HEPES, 1% non-essential amino acids, 1% essential amino acids, 1% Sodium pyruvate, 2-beta-mercaptoethanol, gentamicin, and sodium bicarbonate to restore pH to approximately 7.4.

### Flow cytometry

After 5 days of culture, cell trace-labeled cells in triplicate wells were combined into one well, harvested by centrifugation, and labeled with live/dead discriminator dye (Zombie Yellow, Biolegend) and antibodies for surface markers CD4, CD8, gamma-delta T cell receptor (γδ-TCR), or B cell marker, followed by appropriate secondary labeling antibody if required (Supplementary Table S2 lists antibodies, secondary labels, and suppliers). Data were collected on a BD FACS Aria flow cytometer and analyzed using FlowJo software. Cells were gated on viability dye exclusion, followed by a forward scatter/side scatter profile historically consistent with ruminant lymphocytes. Samples of less than 2,000 cells after live gating were not further analyzed. Lymphocyte populations were plotted against the fluorescent intensity of each antibody in the following pairs: CD4 vs. B cell markers and CD8 vs. γδ-TCR/WC1. Positive marker gates for each subset were determined by cell pools containing all antibodies except the antibody of interest and the isotype of the antibody of interest (fluorescence minus one). The percentage of each subset was analyzed for a decrease in cell membrane proliferation dye as compared to background or no stimulation wells for assessment of proliferation. Data was further analyzed using GraphPad Prism 7 software, fitting two-way ANOVA with Sidak’s multiple comparisons post-test, comparing within groups (TAHD status) effect of well stimulation as compared to background or no stim wells and Dunnett’s multiple comparisons post-test for within well stimulation between groups with groups being the elk TAHD status and treatments being well stimulation (bacterial antigen, +/− controls). Differences between means were considered significant at *p* ≤ 0.05. As there was no difference (*p* > 0.05) in responses to *T. phagedenis, T. pedis, T. denticola,* or *T. medium* antigens used, these results were combined and treated as replicate wells. Due to the low number of samples with grade 2 or 3 lesions, these two lesion categories were combined for statistical analysis.

## Results

### Animals

A total of 183 elk cows were captured over the total course of the study. Among them, 59 were recaptured subsequently, giving the opportunity to collect serum samples at one or more timepoints. The graphical depiction of the study is in Figure 1. Unfortunately, the goal of recapturing an individual multiple times to evaluate adaptive cellular response over time did not occur. Details of animals captured at each time point are listed in Table 1.

**Figure 2:**
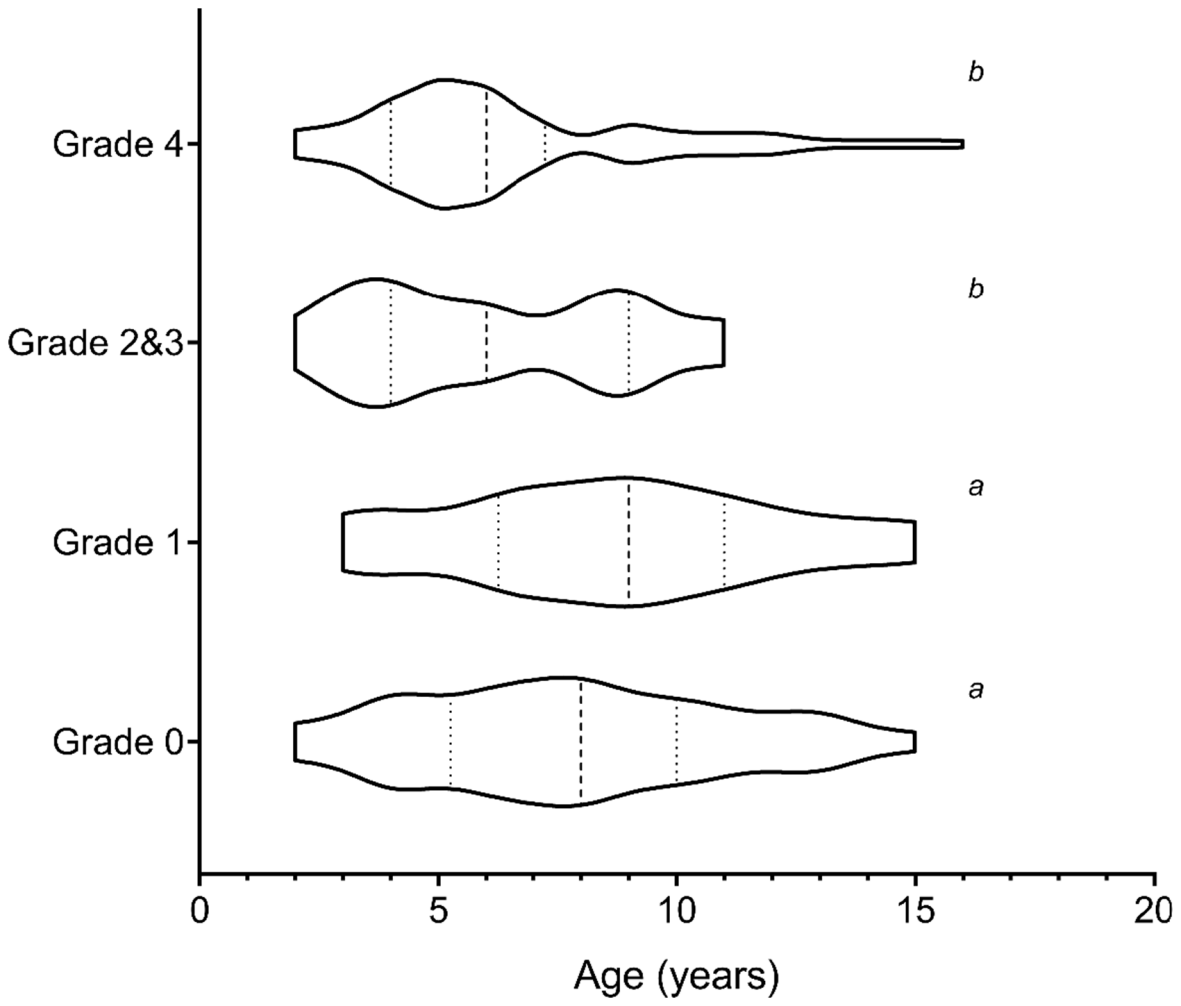
Violin plot showing the age distribution of elk cows live captured and used in the study. The dashed line depicts the median value, and the dotted lines the quartiles. Plots with the same letter are not statistically different from each other but are different from plots with a different letter (*p* < 0.05).

**Figure 3:**
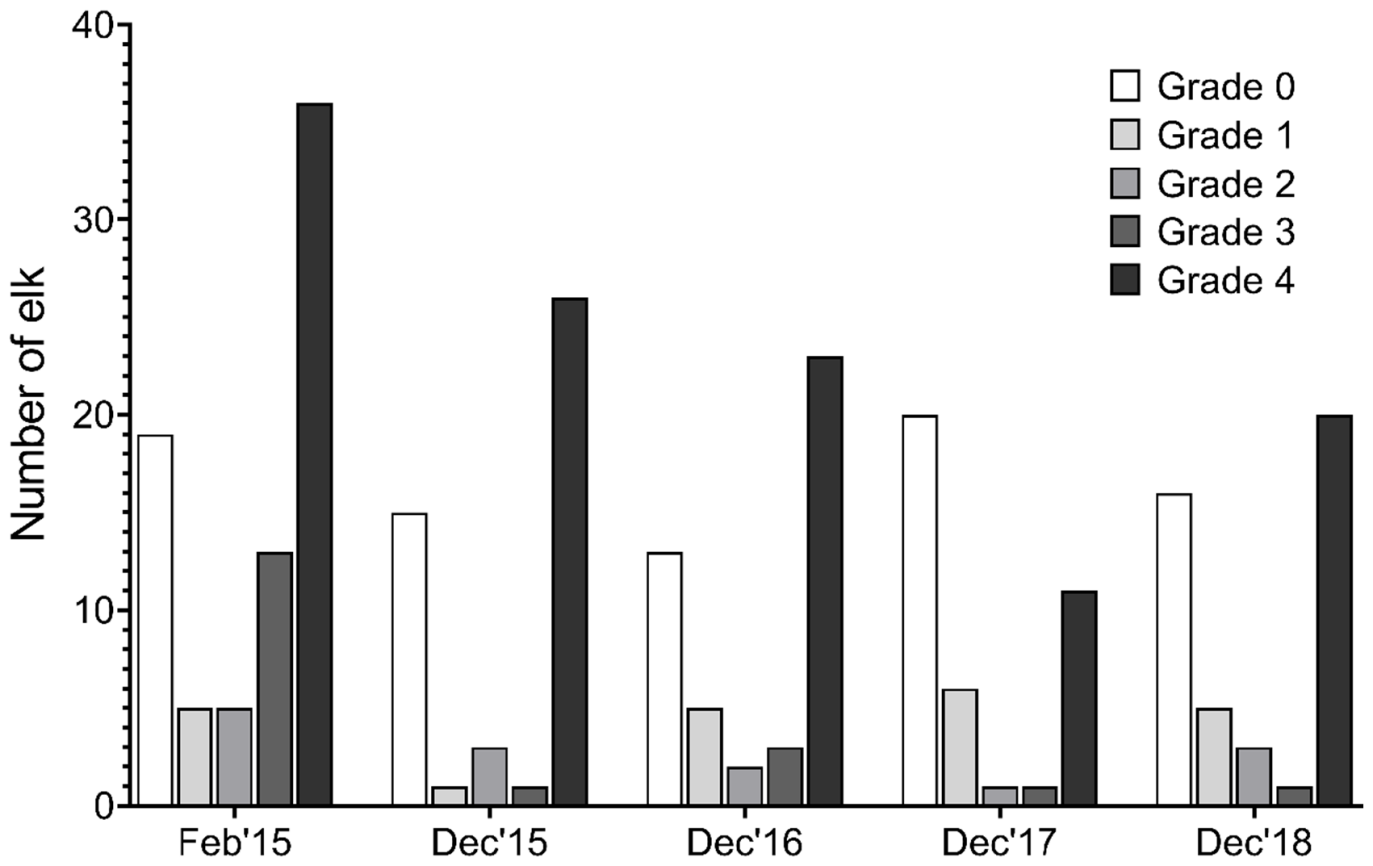
Distribution of lesion grades by capture timepoint.

Within the 59 elk recaptured one or more times in the study, 28 (47.5%) were determined to demonstrate lesions of TAHD+ at the first capture based on visual assessment, 16 (27.1%) demonstrated no lesions at any capture, 13 (22.0%) did not have lesions at the time of the first capture but were considered TAHD+ at a subsequent capture, and 2 (3.4%) animals oscillated between no lesions at first capture and no lesions or grade 1 on subsequent captures. Figure 2 shows the age distribution of animals by lesion grade, with animal age at the time of the last capture used. Grade 4 data demonstrate a lack of normality (*p* < 0.0001) by age and indicate that elk with severe lesions tended to be younger, as compared to the ages observed in other groups. Mean age for elk in grades 0 (healthy) or grade 1 differed (*p* < 0.05) from mean age for elk in grades 2/3 and grade 4, as depicted in Figure 2. The distribution of lesion grades at each capture point was relatively uniform across the 5 years of sampling (Figure 3), but this was most likely influenced by target selection (visibly limping or not-limping elk) and the project goal of maintaining a study population of approximately 66% of animals with TAHD.

### ELISA

**Figure 4:**
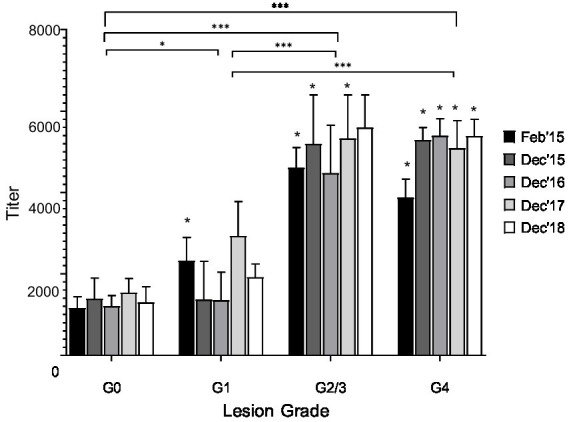
Serum antibody titer to treponemal antigens collected from captured animals grouped by date of capture and lesion grade. Group mean + SEM depicted. Statistical differences within capture date between lesion grades indicated (*, *p* < 0.05). No statistical difference was observed within grades across timepoints. Significant differences between lesion grades without the time variable are shown by brackets (***, *p* < 0.001).

**Figure 5:**
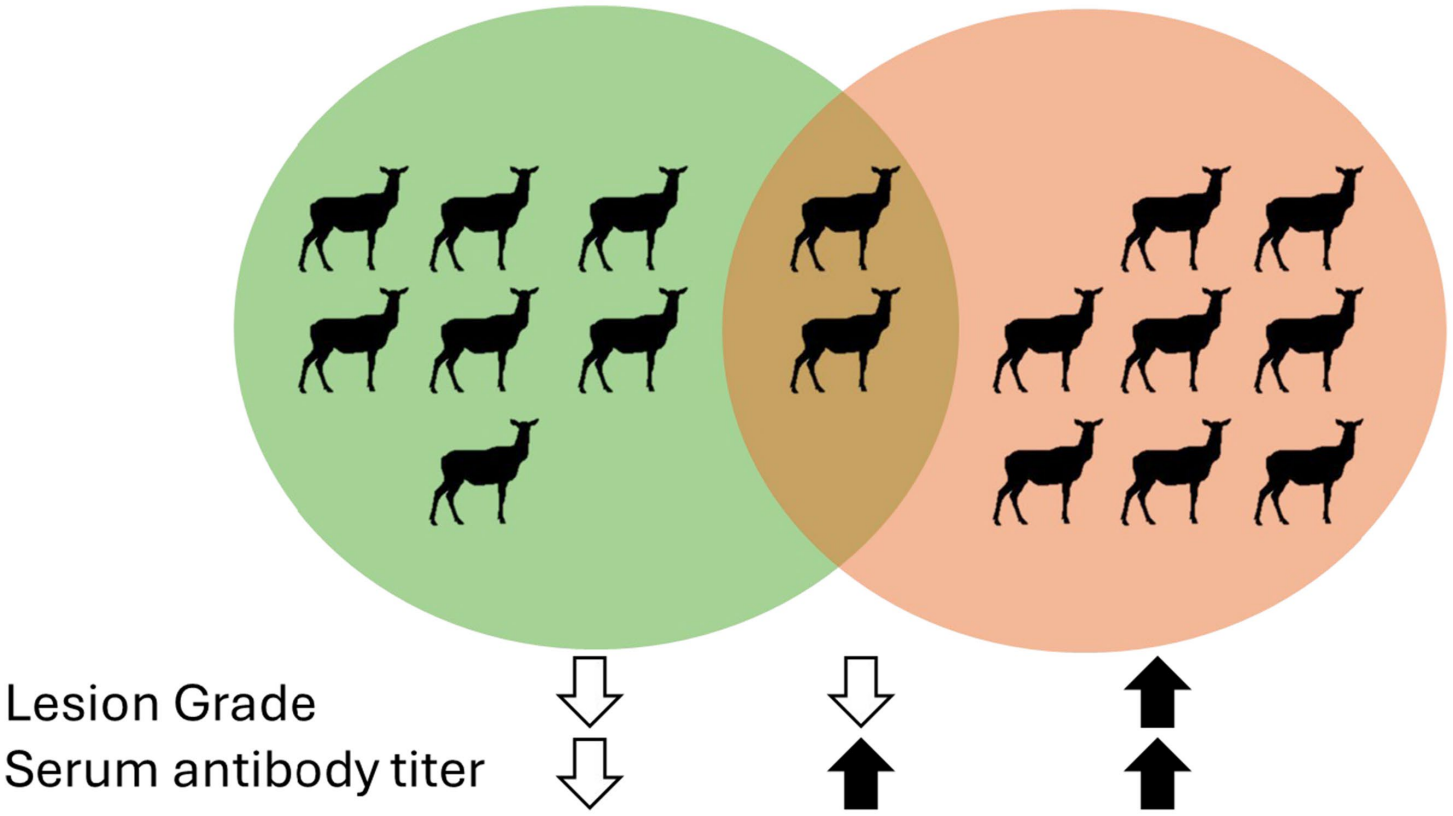
Venn diagram of elk cows captured three or more times over the 5-year study (*n* = 17) grouped for lesion grade and serum antibody titer.

Animals with higher lesion scores (grades 2/3 and 4) had greater (*p* < 0.05) mean serum antibody titers to treponemal antigen when compared to mean antibody titers of elk in grade 0 or 1 groups (Figure 4). The year of sampling did not influence (*p* < 0.05) mean antibody titers. In total, 17 elk were captured multiple times, allowing for antibody titers to be evaluated three or more times over the 5 years (Supplementary file and Supplementary Table S1). Elk with multiple serologic evaluations divide into three groups: (1) negative for lesions and low titers (less than 800) *n* = 7, 42%; (2) negative or grade 1 lesion and high titer (>3,200 on one or more captures) *n* = 2, 12%, and (3) animals with severe lesions (grade 3 or 4) at two or more samplings and high titers (≥6,400) *n* = 8, 48% (Figure 5). Although titers were indicative of exposure and moderately correlated with severity of disease, development of high titers did not correlate with protection from developing TAHD lesions at subsequent captures.

### Lymphocyte proliferation

**Figure 6:**
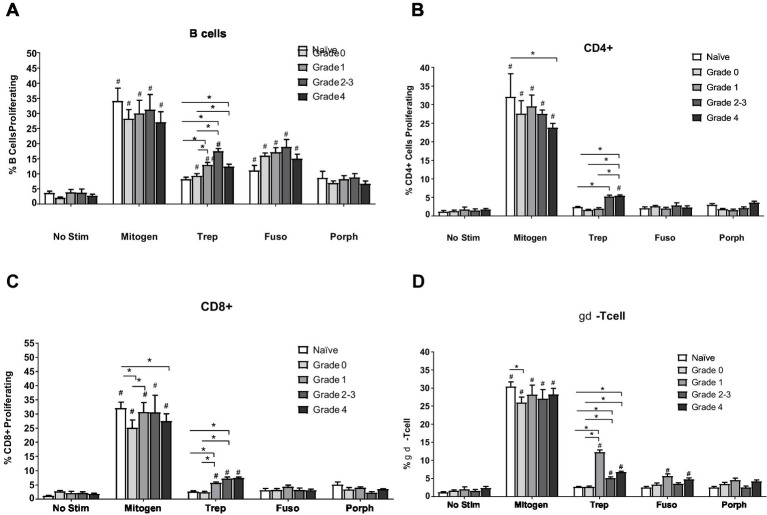
Peripheral blood mononuclear cell proliferation by cell phenotype assayed by flow cytometry. **(A)** B cells (CD21+), **(B)** CD4+ cells, **(C)** CD8+ cells, **(D)** gamma-delta TCR positive T cells. The mitogen used was pokeweed mitogen (10 ug/mL). No stim was media alone; bacterial antigens where whole cell sonicates derived from bacteria associated with TAHD, 5 ug/mL, including mixed *Treponema* (Trep) whole cell sonicate from *T. phagedenis*, *T. pedis*, *T. denticola*, and *T. medium*, *Fusobacterium necrophorum* (Fuso), and *Porphyromonas levii* (Porph). *statistically significant (*p* ≤ 0.05) between lesion grades within stimulation, # statistically significant (*p* ≤ 0.05) within lesion grade from no stim, and bars depict group mean +SEM. No difference was observed between grades 2 and 3, and due to low numbers, these two grades were combined for statistical analysis. Number of animals in each group: naïve *n* = 11, healthy/grade 0 *n* = 17, grade 1 *n* = 9, grade 2/3 *n* = 5, grade 4 *n* = 18.

When PBMCs were stimulated with whole cell antigen from bacteria associated with TAHD lesions (*Treponema* [Trep], *Fusobacterium* [Fuso], and *Porphyromonas* [Porph]), proliferation tended to be greater in groups with more severe lesions, with most cellular subsets demonstrating greater proliferation in response to *Treponema* antigens (Figure 6). The relative total proportion and number of cell types for each lesion grade were roughly the same (Supplementary Figure S1). The percentage of B cells (sIgM+ PBMCs) proliferating in response to *Treponema* antigens was greater (*p* < 0.05) in elk with lesion grades 1 to 4 as compared to the proliferation of B cells from naïve elk or healthy elk in the endemic area (grade 0). All samples demonstrated a greater (*p* < 0.05) percentage of B cells proliferating to *Fusobacterium* antigens, including the naïve elk, possibly indicating some non-specific stimulation; however, samples from elk with grade 1 lesions were statistically different from naïve animals. B cell responses to *Porphyromonas* antigens did not differ (*p* > 0.05) between cells from elk from naïve or healthy endemic populations (Figure 6A). In CD4+, CD8+, and ϒδ populations, greater proliferative responses (*p* < 0.05) in elk from endemic areas and controls were associated with stimulation by *Treponema* antigens (Figures 6B–D). Increased percentages of CD8+ and ϒδ cells from elk in grades 1, 2/3, and 4 had greater percentages of proliferating cells to *Treponema* antigens when compared to elk in the naïve group. In comparison, the percentages of proliferating CD4+ cells were greater (*p* < 0.05) in lesion grades 2/3 and 4, but not grade 1, when compared to responses of naïve elk. There were significant differences in mitogen-stimulated proliferating CD4+ and CD8+ cells between naïve and grade 4 lesion animals and in mitogen-stimulated proliferating CD8+ and ϒδ cells from naïve and grade 0 or healthy elk (Figures 6B–D). Overall, proliferation data of PBMC subsets indicate a trend for greater responses to *Treponema* antigens in animals with more severe lesions, suggesting some development of adaptive cellular responses following infection.

## Discussion

In the data presented, we evaluated humoral and cellular immune responses in wild, free-roaming elk within an area endemic for TAHD. We compared responses of elk with and without TAHD lesions from the same geographic areas and compared them to responses of elk populations considered free of TAHD. Additional data characterizing the epidemiology of TAHD in the endemic area, including survival and health parameters, is published elsewhere (20). Data from the multiyear study suggest that the progression of lesions from healthy animals without lesions to severe lesions (grade 4) and subsequent mortality appears to be rapid, with some animals progressing within months, in between observations at annual captures. The low numbers of grade 2 and 3 lesions further support the hypothesis of rapid lesion progression, with nearly all of the recaptured animals being grade 4 the following capture (Supplementary file). TAHD appears to increase mortality, particularly among elk cows, thereby skewing the age towards younger animals in the higher lesion grades (Figure 2). Longevity of elk cows in a herd may be important for long-term herd survival, not just in producing and successfully raising calves, but also in herd movement between habitats (i.e., up and down mountains, near roads and population centers, open fields vs. wooded cover) in relation to season, including human activities (e.g., hunting seasons) (23).

As has been observed both in elk and cattle with bovine digital dermatitis (BDD), humoral response appears to correlate with severity of lesions but does not appear to contribute to protection from lesion development (1, 12, 15, 16, 24). Serum antibody titers increased with increasing grade but did not differ within grade across the timepoints (Figure 4). In this study, the few elk that were sampled temporally, serum antibody titers increased, but increases were not linear but rather correlated with the pace of disease progression, rapidly increasing once disease was detected (Figure 4; Supplementary file). Perhaps as the severity of the lesion increases, pathogens like *Treponema* are invading further into the tissues, exposing the bacterial antigens to the plethora of inflammatory cells present below the epithelium. Within the study group, only a few animals had high titers but low lesion scores (Figure 5), further supporting this hypothesis. Passive transfer of antibodies may occur, as high serum antibody titers were observed in a previous study in young elk that would have been nursing at the time of sample collection (1). Data from the current multi-year study would suggest that the transfer of maternal humoral immunity dissipates, and young adults are susceptible to lesion development.

Proliferation of B cells was expected, given the strong antibody titers observed in infected animals. While proliferation was detected in T cells (CD4+ and CD8+) in response to treponemal antigen, it was greater in TAHD+ elk than in naïve or grade 0 elk; the magnitude of the response was low (less than 10%) (Figure 6). While not unusual, the magnitude of responses is comparable to antigen-specific responses to other bacterial infections in bovines (25). Relative numbers of each respective cell type were the same for all groups (Supplementary Figure S1). Responses are also comparable to or greater than those observed in BDD infection, both naturally occurring and repeated experimental exposure (15, 16). Our observation of increases in antigen-specific ϒδ-T cell responses to treponemal antigens in grade 1 animals is intriguing. Similar increases in proliferative responses were observed in cattle demonstrating BDD lesions (15) and also in cattle infected with another spirochete bacterium, *Leptospira borgpetersenii* (25). The bovine WC1 receptor on ϒδ-T cells has a high affinity for bacterial antigens, including leptospiral ligands (26). The exact role of ϒδ-T cells in disease protection is unknown, but their multifunctional plasticity in ruminants suggests potential roles in both early responses to infection and development of memory-like cells with effector cell functions (27). The effector functions of ϒδ-T cells in ruminants can vary widely and include proinflammatory secretion of IL-17 or regulatory-like function with production of IL-10 (25, 27, 28). Further study is needed in ruminant models of DD to determine the role these early ϒδ-T cells play in protection or perpetuation. While cattle develop serum antibody responses to *Treponema* and other pathogens found in DD lesions, antigen-specific cellular responses appear to be limited and short-lived (16, 24). One hypothesis is that during chronic infection, the adaptive immune response may be suppressed to prevent inflammatory responses as observed with resident intestinal bacteria (29). Or the lack of CD4+ cell response could represent exhaustion, as evidenced in other long-term chronic viral or parasitic infections (30). However, a more probable hypothesis is that antigen responsive cells traffic to sites of infection and are not represented within the peripheral circulation. In sheep and cattle, ϒδ-T cells home to the skin and draining lymph nodes following increased surface expression of CD62L and skin-homing receptors CCR4 and CCR10 (31, 32). Further study is needed to determine if circulating ϒδ-T cells in elk or cattle with acute *Treponema* lesions have increased expression of CCR4 and CCR10 or if these cells can be detected in lesions or lymph nodes draining the site of infection.

Nutritional status is thought to influence immunological function and disease susceptibility of animals. While the nutritional status of the elk in southwest Washington state is generally poor (33), animals with TAHD may experience increased nutritional and other stressors. This may partially explain the lower mitogenic proliferation observed in CD4+ and CD8+ T-cell responses compared to naïve elk from the Yakima Valley, which receive additional winter forage or NADC and are housed under optimal conditions. While numerically significant, these responses are biologically small, and we would be hesitant to claim that there is severe immunosuppression in the TAHD-infected elk. The mitogen proliferative responses for CD8+ and ϒδ-T cells were increased in naïve elk over grade 0 or healthy elk from the endemic area, again indicating there may be a role for enhanced nutrition and lack of reproductive stress in the NADC elk (Figures 6B–D). Much like pregnancy, immune functions seem to be prioritized in wildlife, or at least in elk, and are maintained despite stressors such as nutrition and overcrowding (34). Many studies of *Treponema* disease, including hoof disease and periodontal disease, have reported strong inflammatory responses at the site of infection (4, 35–38). It is theorized that these periodontal pathogen-activated inflammatory macrophages and T cells, which circulate and migrate to other tissues, contribute to hypertension, diabetes, and other heightened host inflammatory conditions. Thus, we were surprised to not see reactive peripheral lymphocytes, even though in cattle with DD there are no clinical signs of disease (fever, malaise, etc.) other than the lameness and localized skin lesions.

A major limitation of the current study was the sample size. In cattle with digital dermatitis, peripheral immunological responses are minimal and short-lived, suggesting cells may be localized to infected tissue (16, 24). The major objectives of the current study by WDFW were to characterize survival, calf rearing, and disease progression within elk populations endemic with TAHD. Thus, we did not want to collect invasive samples, such as biopsies, which would create an open wound for bacterial entry or otherwise alter the disease status of the hoof. Therefore, immunological evaluation was limited to blood sampling and characterization of circulating lymphocytes.

Another major limitation of the study is related to the numerous limitations of working with wildlife and live animal capture. Time constraints for collecting and shipping samples (shipping samples before noon on the day of collection), limitations related to when samples could be collected (after hunting season but before winter), weather, budgetary, and personnel restrictions all influenced project objectives retrospectively since the number of animals and desired specimens/individuals could not always be obtained. Although the project originally intended to sample individual elk on sequential years to evaluate cellular immune responses over time, this was found to not be practical. For example, one elk female was first captured in February 2015 and recaptured in December 2015, too late in the day’s sequence for blood could not be shipped for cellular immunology; she was not recaptured at later time points despite signals from the collar indicating survival for several more years. Although serum samples were obtained on multiple samplings from 53 of 184 animals, limitations related to sample collection and shipping led to cellular immune responses being characterized only once for each elk during the 5-year study.

An issue for any study with wildlife is the limitations in reagents that have been identified for use in those species. Although antibodies for use in flow cytometry panels characterizing the major T cell subsets within elk (*Cervus canadensis*) are available, similar reagents for detecting cytokines are lacking. Thus, effector functions of T cell responses could not be characterized at that time. Additional understanding of the functionality of proliferating cells would have been useful to help assess whether the immune responses we detected might provide *in vivo* benefits in responding to TAHD lesions. With the availability of the elk genome (39) and continued research into elk cellular immune responses, hopefully, additional tools will be available in the future to provide additional insights into functional immune responses in elk to *Treponema* and other bacterial infections.

## Conclusion

Elk with TAHD produced a robust serum antibody to treponeme antigens that correlated with lesion severity, providing further support for identifying these bacteria as key pathogens in TAHD lesions. Lymphocyte proliferative responses were primarily limited to treponemal antigens for CD4+ and CD8+ T cells and were greatest in elk with more severe lesions. Our data indicate the development of an adaptive immune response to *Treponema* in elk, as evidenced by TAHD lesions. As we observed some responses in ϒδ-T cells in early lesions, further study is warranted to elucidate the role of these cells in TAHD lesion development.

## Data Availability

The original contributions presented in the study are included in the article/Supplementary material, further inquiries can be directed to the corresponding author.

## Supplementary material

SUPPLEMENTARY FIGURE S1 Number and percentages of lymphocytes as analyzed by flow cytometry after culture for 5 days in the presence of bacterial antigens. Mitogen used was pokeweed mitogen (10ug/mL), No Stim was media alone, bacterial antigens were whole cell sonicates derived from bacteria associated with TAHD, 5ug/mL, including mixed *Treponema* (Trep) whole cell sonicate from *T. phagedenis*, *T. pedis*, *T. denticola* and *T. medium*, *Fusobacterium necrophorum* (Fuso) and *Porphyromonas levii* (Porph). * statistically significant (*p* ≤ 0.05) between lesion grades within stimulation, # statistically significant (*p* ≤ 0.05) within lesion grade from No Stim, Bars depict group mean +SEM. Due to low numbers, grades 2 and 3 were combined for statistical analysis. Number of animals in each group: naïve *n* = 11, healthy/grade 0 *n* = 17, grade 1 *n* = 9, grade 2/3 *n* = 5, grade 4 *n* = 18. **(A)** Total number of lymphocytes analyzed averaged per group. Minimum of 2000 live lymphocytes were needed to be included in analysis. Sample analysis was stopped at 100,000 live lymphocytes. **(B)** Percentage of B cells (CD21+) in lymphocyte gate per well stimulation and lesion grade. **(C)** Percentage of CD4+ cells in lymphocyte gate per well stimulation and lesion grade. **(D)** Percentage of CD8+ cells in lymphocyte gate per well stimulation and lesion grade. **(E)** Percentage of ϒδ-TCR+ cells in lymphocyte gate per well stimulation and lesion grade.
