# Supplementary figures and images for: Immunological response of live-captured wild elk (Cervus canadensis) to Treponeme-Associated Hoof Disease antigens
Source: Front Vet Sci. 2026 Feb 5;12:1652577. doi: 10.3389/fvets.2025.1652577 (PMC12917888; doi:10.3389/fvets.2025.1652577)

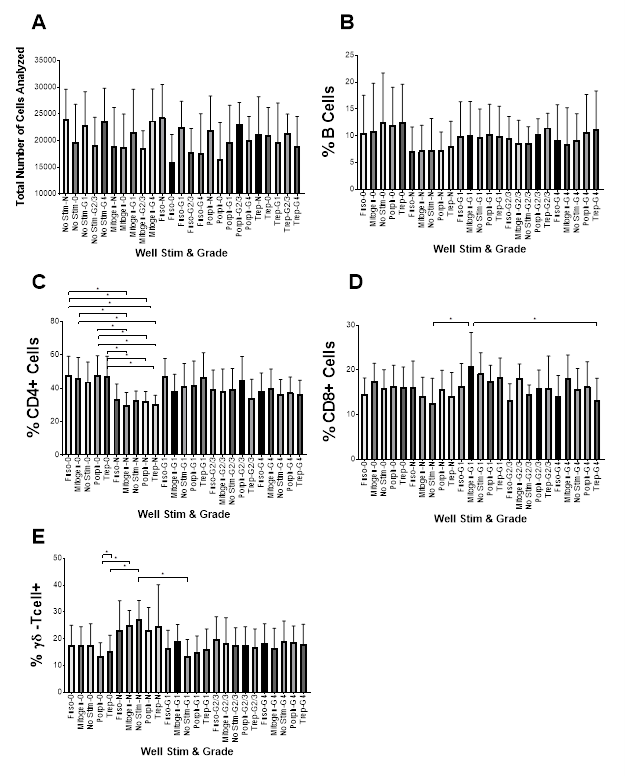

Supplement: SUPPLEMENTARY FIGURE S1 — Number and percentages of lymphocytes as analyzed by flow cytometry after culture for 5 days in the presence of bacterial antigens. Mitogen used was pokeweed mitogen (10ug/mL), No Stim was media alone, bacterial antigens were whole cell sonicates derived from bacteria associated with TAHD, 5ug/mL, including mixed Treponema (Trep) whole cell sonicate from T. phagedenis, T. pedis, T. denticola and T. medium, Fusobacterium necrophorum (Fuso) and Porphyromonas levii (Porph). * statistically significant (p ≤ 0.05) between lesion grades within stimulation, # statistically significant (p ≤ 0.05) within lesion grade from No Stim, Bars depict group mean +SEM. Due to low numbers, grades 2 and 3 were combined for statistical analysis. Number of animals in each group: naïve n = 11, healthy/grade 0 n = 17, grade 1 n = 9, grade 2/3 n = 5, grade 4 n = 18. (A) Total number of lymphocytes analyzed averaged per group. Minimum of 2000 live lymphocytes were needed to be included in analysis. Sample analysis was stopped at 100,000 live lymphocytes. (B) Percentage of B cells (CD21+) in lymphocyte gate per well stimulation and lesion grade. (C) Percentage of CD4+ cells in lymphocyte gate per well stimulation and lesion grade. (D) Percentage of CD8+ cells in lymphocyte gate per well stimulation and lesion grade. (E) Percentage of ϒδ-TCR+ cells in lymphocyte gate per well stimulation and lesion grade. [file Image_1.tif]
