# Supplementary material for: Immunological response of live-captured wild elk (Cervus canadensis) to Treponeme-Associated Hoof Disease antigens
Source: Front Vet Sci. 2026 Feb 5;12:1652577. doi: 10.3389/fvets.2025.1652577 (PMC12917888; doi:10.3389/fvets.2025.1652577)
Supplement: Supplementary file 3 [file Table_1.docx]

| Table S1: Numbers of female elk per lesion category per timepoint used in ELISA assays | | | | | |
| --- | --- | --- | --- | --- | --- |
|  | **Feb'15** | **Dec'15** | **Dec'16** | **Dec'17** | **Dec '18** |
| Naïve |  |  |  | 2 | 3 |
| Grade 0 | 19 | 15 | 13 | 20 | 17 |
| Grade 1 | 5 | 1 | 4 | 6 | 5 |
| Grade 2/3 | 17 | 4 | 5 | 2 | 4 |
| Grade 4 | 33 | 26 | 23 | 11 | 20 |
