# Supplementary material for: Immunological response of live-captured wild elk (Cervus canadensis) to Treponeme-Associated Hoof Disease antigens
Source: Front Vet Sci. 2026 Feb 5;12:1652577. doi: 10.3389/fvets.2025.1652577 (PMC12917888; doi:10.3389/fvets.2025.1652577)
Supplement: Supplementary file 4 [file Table_2.docx]

| Table S2: Antibodies used in Flow Cytometry | | | | |
| --- | --- | --- | --- | --- |
| Marker | Source | Clone | Secondary | Source |
| CD4 | WSU* | 17D1 | PE TXRD-αIgG1 | Southern Bio-0102-13 |
| CD8 | WSU | ST8 | APC-αIgM | Biolegend-406509 |
| gdTCR (TCR1 δ chain) | WSU | GB21A | APC-Cy7-αIgG2b | Southern Bio-1090-19 |
| CD21 | WSU | Baq15A | PE-direct conjugated in house |  |
| *WSU Washington State University Monoclonal Antibody Resource Center | | | | |
